# Thoracic surgery in the non-intubated spontaneously breathing patient

**DOI:** 10.1186/s12931-022-02250-z

**Published:** 2022-12-27

**Authors:** Matthias Grott, Martin Eichhorn, Florian Eichhorn, Werner Schmidt, Michael Kreuter, Hauke Winter

**Affiliations:** 1 Department of Thoracic Surgery, Thoraxklinik, University Hospital Heidelberg, Roentgenstrasse 1, 69126 Heidelberg, Germany; 2 Translational Lung Research Centre Heidelberg (TLRC-H), German Centre for Lung Research (DZL), Heidelberg, Germany; 3 Center for Interstitial and Rare Lung Diseases, Pneumology Thoraxklinik, University Hospital Heidelberg, Roentgenstrasse 1, 69126 Heidelberg, Germany; 4 Department of Anaesthesiology and Intensive Care Medicine, Thoraxklinik, University Hospital Heidelberg, Roentgenstrasse 1, 69126 Heidelberg, Germany

**Keywords:** Non-intubated video assisted thoracic surgery, Lung cancer, Interstitial lung disease

## Abstract

**Background:**

The interest in non-intubated video-assisted thoracic surgery (NIVATS) has risen over the last decade and numerous terms have been used to describe this technique. They all have in common, that the surgical procedure is performed in a spontaneously breathing patient under locoregional anaesthesia in combination with intravenous sedation but have also been performed on awake patients without sedation. Evidence has been generated favouring NIVATS compared to one-lung-ventilation under general anaesthesia.

**Main body:**

We want to give an overview of how NIVATS is performed, and which different techniques are possible. We discuss advantages such as shorter length of hospital stay or (relative) contraindications like airway difficulties. Technical aspects, for instance intraoperative handling of the vagus nerve, are considered from a thoracic surgeon’s point of view. Furthermore, special attention is paid to the cohort of patients with interstitial lung diseases, who seem to benefit from NIVATS due to the avoidance of positive pressure ventilation. Whenever a new technique is introduced, it must prove noninferiority to the state of the art. Under this aspect current literature on NIVATS for lung cancer surgery has been reviewed.

**Conclusion:**

NIVATS technique may safely be applied to minor, moderate, and major thoracic procedures and is appropriate for a selected group of patients, especially in interstitial lung disease. However, prospective studies are urgently needed.

## Introduction/background

With the introduction of minimally invasive surgical techniques in the early 1990s, video-assisted thoracic surgery (VATS) under general anaesthesia (GA) with one-lung ventilation (OLV) advanced to become the standard procedure in lung surgery [1]. Due to the use of a double lumen tube (DLT) and positive pressure ventilation, OLV can be associated with intubation-related airway trauma and barotrauma of the lung which can lead to severe long-term damage to the lung parenchyma. This can be avoided by employing thoracic surgery procedures performed in non-intubated, spontaneously breathing patients (NIVATS) [2].

Pompeo et al. [3] demonstrated that awake thoracoscopic resections of pulmonary nodules are feasible and as safe as surgery under GA with OLV.

Numerous terms have been used to describe video-assisted thoracic procedures in awake patients such as non-intubated video-assisted thoracic surgery (NIVATS) [2], awake video-assisted thoracic surgery (AVATS) [4], non-intubated thoracic surgery (NITS) [5], monitored anaesthesia care thoracic surgery (MACTS) [1], awake thoracic surgery (ATS) [6], spontaneous-ventilation video-assisted thoracoscopic surgery (SV-VATS) [7] or “tubeless” [8].

They all have in common, that the surgical procedure is performed on a non-intubated spontaneously breathing and usually sedated patient in combination with a locoregional anaesthetic technique but have also been performed on awake patients without sedation [9]. In recent years, the NIVATS technic has become more and more established in Europe, so that now not only small minimal invasive procedures but also large minimal invasive surgeries are performed. In surveys conducted in Europe among members of the European Society of Thoracic Surgeons (ESTS), 62 of 105 thoracic surgeons (59%) and 42% of thoracic surgeons in the German Society of Thoracic Surgery (DGT) reported performing non-intubated thoracic procedures, particularly in patients with poor cardiopulmonary function or for the diagnosis of interstitial lung disease [5, 10].

## NIVATS procedure

### Locoregional anaesthesia techniques

Local anaesthetic techniques essential for NIVATS include (1) thoracic epidural anaesthesia (TEA), (2) cervical epidural anaesthesia (CEA), (3) paravertebral block (PVB), (4) intercostal (nerve) block (ICB), (5) injection of local anaesthetic (LA) at the site of trocar incision (6) local vagus blockade, (7) erector spinae plane block (ESPB), (8) serratus anterior plane block (SAPB) and (9) pectoralis nerve block (PECS).

TEA/CEA is used to achieve a somatosensory and motor block of the intercostal muscles at C7 to T8 while preserving diaphragmatic breathing. Horner syndrome may develop if C6 is impaired. After premedication with oral midazolam (0.1 mg/kg, usually 7.5 mg tablet), the epidural catheter is inserted with a low resistance syringe. The loss of resistance and hanging drop methods are used to localize the epidural space [11]. A successful level of anaesthesia is tested either by pin-prick sensation or warm-cold discrimination.

In the operating room, the applied TEA/CEA catheter used is equipped with ropivacaine 0.5% ± sufentanil (1.66 µg/ml) with continuous or fractionated administration of local anaesthetics. At the end of surgery, the dosage of the local anaesthetics is usually reduced [3, 11].

Injection of ropivacaine for PVB is usually performed under ultrasound or nerve stimulator guidance, but can also be performed with the commonly used technique of resistance loss [12]. The choice of site depends on the nature of the procedure and the planned incision location. PVB can be performed bilaterally if necessary (e.g., NUSS surgery in children) [13].

A Cochrane Review by Yeung et al. [14] compared the two regional techniques with respect to the analgetic effect, minor and major complications, length of hospital stay and cost effectiveness. They included all randomized controlled trials (RCTs) comparing TEA with PVB in patients operated on for lung surgery with thoracotomy. They found a similar effectiveness in pain reduction with reduced risk for minor complications in patients treated with a PVB.

D’Ercole et al. [15] demonstrated lesser effectiveness of thoracic PVB compared to TEA for therapeutic effectiveness of postoperative analgesia causing fewer side effects for unilateral and bilateral thoracic surgery. PVB anaesthesia may be used to avoid temporary contralateral sympathectomy, thereby minimizing hypotension.

Liang et al. [16] found a disadvantage of PVB in relieving postoperative pain in the first 24 h after surgery. Patients required higher opioid concentrations to adequately relieve postoperative pain but had a lower incidence of hypotension.

For applying an ICB a 50:50 mixture of a short- (e.g., lidocaine) and long-lasting (e.g., bupivacaine) LA is injected beneath the parietal pleura and along the intercostal space and trocar site(s) [17, 18]. It can also be injected under thoracoscopic guidance directly under the parietal pleura, 2 cm lateral to the sympathetic nerve [18].

Regional anaesthesia techniques may provide better hemodynamic stability, superior postoperative analgesia, decreased surgical stress response with fewer side effects, such as nausea and vomiting, if compared to GA [12]. Adequate analgesia is mandatory and required for maintenance of constant intraoperative verbal interaction with the patient during surgery for reassuring his wellbeing [19]. It is important to wait at least 20 min after administration of TEA/CEA/PVB/ESPB/SAPB/PECS before starting surgery to reduce the pain stimulus, especially if combined with sedation.

Another regional anaesthesia technique is the erector spinae plane block (ESPB) [20, 21], which may also be combined with PVB [22]. Under ultrasound guidance a nerve block needle is positioned between T5 to T8 level over the transverse process and beneath the erector spinae muscle to reach the interfascial space where local anaesthetics (e.g., 10 ml of 0.5% bupivacaine and 5 ml of 2% lidocaine or 0.375% ropivacaine mono) are injected caudally to cranially after hydro dissection with normal saline [19–21]. Successful blockade is verified by a pinprick test.

Furthermore, serratus anterior plane block (SAPB) may be an option for regional anaesthesia. Under ultrasound guidance local anaesthetics (e.g. 20–25 ml of 0.125–0.25% levobupivacaine) are injected between the serratus anterior and the intercostal muscles in the midaxillary line/5th rib thus blocking the lateral cutaneous branches of the T2–T9 spinal neurons [23, 24].

The pectoralis nerve block (PECS) is performed with the patient in supine position and ultrasound guidance. The coracoid process is visualized, and regional anaesthesia is performed by injecting 0.2 ml/kg of 0.25% bupivacaine or 0.5% ropivacaine into the interfascial plane between the pectoralis major and minor muscles [25].

### Complementary sedation

TEA/CEA/ICB//PVB/ESPB/SAPB/PECS/LA are usually combined with propofol ± remifentanil or dexmedetomidine to induce mild sedation for the patient’s comfort and anxiolysis [18, 19, 22, 26–28]. A bispectral index sensor (BIS) can be placed on the patient’s forehead to measure the intraoperative state of consciousness with a target value between 40 and 60 [7, 18, 27, 29]. Low-volume music contributes to a comfortable environment and may reduce the patient’s anxiety [19, 30].

However, sedation is not mandatory. Elia et al. performed bilateral sympathectomies in 15 awake patients without sedating medications or additional intravenous analgesia [31]. Pompeo et al. [32] reported that mild intraoperative sedation was required to treat panic attacks in only two of 21 patients with spontaneous pneumothorax recurrence who underwent triportal bullectomy and pleurectomy. Numerous combinations of locoregional anaesthesia and sedation are possible. To date, no standard operating procedures (SOPs) have been proposed. Most surgeons prefer to sedate the patient during surgery to reduce patient movement and discomfort, especially for longer, more complex surgeries [33].

### Airway management

A laryngeal mask may be used for assisting airway management [27, 28]. Other possible options for oxygen supply during surgery are breathing through a ventilation mask [18] or providing oxygen with a casual oxygen mask. Kiss et al. [9] used a high-flow oxygen mask (15 L O_2_/min) in high risk patients undergoing thoracic procedures (pleurectomy for recurrent pneumothorax, pleurostomy, emphysema surgery, lung biopsy) to prevent desaturation.

After thoracotomy, iatrogenic pneumothorax develops under spontaneous breathing, leading to a decrease in lung volume and thus to pendulum breathing. Rebreathing of exhaled gases (“carbon dioxide rebreathing”) causes hypercapnia [34, 35]. In addition, mediastinal displacement may occur, causing compression of the non-operated lung (= dependant lung) and further impairing lung function [34]. Transient tachypnoea may occur as a compensatory mechanism for hypercapnia. Tachypnoea often resolves spontaneously after a few minutes and should not be treated with deeper sedation [35, 36]. Permissive hypercapnia (PaCO_2_ < 55 mmHg) during NIVATS can be tolerated [37] because it usually resolves spontaneously postoperatively [34, 35, 38]. Dong et al. [39] determined PaO_2_ and PaCO_2_ values preoperatively, as well as intraoperatively immediately before and 15, 30, and 60 min after wound closure. They observed stable PaO_2_ values and a gradual increase in PaCO_2_ values with normalization within 1 h after wound closure. In patients with rapid-onset and severe respiratory failure secondary to iatrogenic surgical pneumothorax, a chest drain connected to a negative pressure of − 15 cmH_2_O can accelerate re-expansion of the collapsed lung helping to gain time for trouble shooting. Negative pressure can also help detect an air leak at the end of the operation.

Respiratory reasons for a conversion from NIVATS to GA are persistent hypoxemia (PaO_2_ < 60 mmHg or SpO_2_ < 90%) [40], severe hypoxemia (PaO_2_ < 55 mmHg or SpO_2_ < 85%), severe hypercapnia (PaCO_2_ > 70–80 mmHg) and acidosis (pH < 7.1) [41].

For safety reasons, a single-lumen endotracheal tube should always be prepared for emergency situations [42] and a large (transparent) surgical drape should be kept on hand for temporary wound coverage. The trocar incisions can quickly be closed with a single stitch [6]. A bronchoscope should be at hand to have a safe access to the bronchial system [43]. The anaesthesiologist should be skilled in intubating a patient in the lateral decubitus position.

### Surgical procedures

Many different surgical procedures can be performed under spontaneous ventilation as reviewed by Gonzalez-Rivas et al. [41] and Kiss et al. [6], ranging from therapeutic drainage of pleural effusion up to complex anatomical resections such as segmentectomies, lobectomies, pneumonectomies or tracheal resections. At the end of the operation after reclosure of the thoracotomy the patient is asked to cough after chest tube insertion in order to re-expand the lung [18].

Surgical times from skin incision to wound closure reported in the literature vary depending on the extent of the surgical procedure. Ambrogi et al. [44] reported a mean operative time of 38 min for minor thoracic procedures such as NIVATS biopsies or NIVATS decortication for pleural empyema. Tacconi et al. [37] reported a mean operative time of 50 min for complex pleural effusions. Chen et al. [40] published a mean operative time of 161.9 min for NIVATS lobectomies, and Jiang et al. [7] reported a mean operative time of 162.5 min for tracheal resections. A meta-analysis published by Zhang et al. [45] in 2021, which included 14 randomized controlled trials involving 1426 patients, found no statistically significant differences in mean operative times between intubated and non-intubated patients.

NIVATS can also be performed in children. Wei et al. [13] conducted an RCT to investigate whether non-intubated anaesthesia combined with PVB can improve postoperative recovery compared with general anaesthesia. The total study cohort of 60 children aged 3 to 8 years was divided into two groups of 30 patients each who underwent VATS for surgical correction of pectus excavatum (NUSS procedure), mediastinal tumour resection, or lung biopsy. One child in each group was excluded from the data analysis. In the intubated group, one child had to be converted to thoracotomy because of excessive bleeding. In the non-intubated group, one child required mechanical ventilation because of increasing CO_2_ retention (up to 82 mmHg) and hypoxemia (SpO_2_ < 92%). The length of hospital stay was 4 days in non-intubated children and significantly shorter than compared to 5 days in the anesthetized children. Spontaneously breathing children required less time from complete unconsciousness to full awakening, a shorter stay in the post-anaesthesiology care unit (PACU) and had a lower incidence of emergency delirium. Median time to first feeding, mobilization, and pain intensity were also significantly lower. Surgical outcome was comparable in both groups. Ventilated children complained significantly more often of respiratory symptoms such as hoarseness, sore throat, and irritable cough after intubation.

### Contraindications

There are anaesthesiologic and surgical contraindications for NIVATS. Locoregional anaesthetic techniques in spontaneously breathing patients should not be used in patients with a difficult airway, as an intraoperative conversion to GA might be challenging and troublesome [12]. Common contraindications for applying locoregional anaesthesia include allergy to local anaesthetics, coagulation disorders, active neurologic disorders and skin infections at the site of TEA/CEA insertion [11]. Patients with an ASA score higher than 3, sleep apnoea, or unfavourable spinal anatomy [18], as well as uncooperative patients [46] or patients with an existing language barrier, may not be suitable for awake anaesthesia procedures. However, Kiss et al. [9] were able to safely perform a NIVATS procedure in four patients with an ASA score of 4 (three patients with pneumothorax and one patient with pyothorax). Table 1 summarizes the contraindications to NIVATS.

**Table 1:** Contraindication to non-intubated video-assisted thoracic surgery

Anaesthesiologic contraindications	Surgical contraindications	Relative contraindications	Others
Allergy to local anaesthetic agents	Extensive adhesions obliterating the pleural space	Congestive heart failure	Active neurologic/severe psychiatric disorders
Coagulation disorders	Prior talc pleurodesis	Mitral-/aortic valve stenosis	Prior radiation treatment for thoracic malignancy
Skin infection at the site of TEA/CEA/PVB/ESPB/SAPB/PECS	Previous thoracotomies	Obstructive cardiomyopathy	Morbid obesity
Unfavourable spinal anatomy	Extensive pleural diseases	Difficult airway	Atrial carbon dioxide tension > 55 mmHg
Markedly unstable or shocked patients		ASA score higher than 3	
Inability to tolerate single-lung ventilation		Sleep apnoea	

*TEA* thoracic epidural anaesthesia, *CEA* cervical epidural anaesthesia, *PVB* paravertebral block, *ESPB* erector spinae plane block, *SAPB* serratus anterior plane block, *PECS* pectoralis nerve block, *ASA* American Society of Anaesthesiologists Score

### Ventilator associated problems

Endotracheal intubation may cause local complications such as airway hyperresponsiveness, postoperative sore throat and major airway and vocal cord injury [47, 48]. Mechanical lung ventilation may cause serious side effects, such as airway pressure-induced lung injury and lung damage by overdistension and sheer stress of repetitive alveolar collapse [49, 50]. OLV with large tidal volumes may contribute to postoperative acute lung injury (PALI). Patients undergoing pneumonectomy and patients with decreased respiratory function are particularly at risk [51, 52]. OLV may promote the production and release of proinflammatory substances in the alveoli of the dependent (= non-operated) lung [53]. Liu et al. [54] corroborate these findings in their randomized controlled trial (RCT). They tested and compared bronchoalveolar lavage fluid before and after bullectomy in intubated and awake patients for concentrations of tumour necrosis factor (TNF)-α and high-sensitivity C-reactive protein (hs-CRP). They found lower levels of inflammatory cytokines in awake patients. A similar observation was reported by Jeon et al. [55] who found significantly lower systemic levels of interleukin (IL-)6 and TNF-α in non-intubated patients with early-stage lung cancer within the first 24 h after minimal invasive lobectomy.

### Avoiding general anaesthesia

The need for deep sedation and muscle relaxation of patients undergoing GA has notable negative side effects. Intraoperatively low mean arterial pressure (MAP) and deep hypnosis—measured by low Bispectral Index (BIS) scores in combination with low minimal alveolar concentration (MAC) of volatile anaesthetics are associated with a prolonged length of hospital stay and an increased 30-day mortality [56]. Muscle relaxants may contribute to a diaphragmatic dysfunction increasing the risk of lung atelectasis in the non-operated lung and may promote a right-to-left shunt aggravating hypoxemia. This increases the risk for the patient to be ventilated postoperatively in case of a residual neuromuscular blockade [6, 57]. Intravenous analgesics, especially opioids, may cause postoperative anaesthesiologic complications such as hyperalgesia, nausea and/or vomiting and ventilatory depression [49] which could be a reason for longer duration of anaesthesia [58].

Regional anaesthesia eliminates GA-related side effects, possibly resulting in a lower operative risk and shorter hospital stay, particularly in older patients and in those with compromised respiratory function [19, 34]. A disadvantage of NIVATS is preserved diaphragmatic function [1] and intraoperative diaphragmatic movement, which may lead to unsafe surgery or conversion to GA [7, 55]. Therefore, NIVATS should be avoided for the treatment of lesions that are close to or infiltrate the diaphragm.

### Technical aspects

To date, multiple meta-analyses (see below) were able to show significant differences between DLT-VATS and NIVATS for thoracic surgery favouring NIVATS. Observed advantages of NIVATS were a shorter operative time, shorter chest tube indwelling time, and less intraoperative and postoperative blood loss compared with DLT-VATS.

Shi et al. [59] performed a meta-analysis of 1138 patients including ten studies (3 RCTs, 7 observational studies) comparing postoperative pulmonary complications (PPC) between non-intubated and intubated patients undergoing lung resections. They analysed the postoperative incidence of atelectasis, pneumonia, respiratory failure, bronchospasm, aspiration, pneumonitis, pleural effusion, and pneumothorax. PPC were similar in the DLT-VATS and the NIVATS groups. Of note, the NIVATS patients had a significantly shorter length of hospital stay.

Another meta-analysis by Zhang et al. [60] included 15 studies (5 RCTs, 10 retrospectives) comparing NIVATS to DLT-VATS procedures with a total of 1684 patients. They analysed postoperative surgical and anaesthesiologic complications such as hoarseness, haemothorax, cardiac complications, persistent air leakage > 5 days and perioperative mortality rate. They found that complications were significantly less frequent in NIVATS patients. With regard to secondary endpoints such as total operative time, anaesthesia time, length of hospital stay, chest tube indwelling time, and postoperative pain level, the NIVATS procedure was proven to be beneficial. A subgroup analysis of patients after NIVATS or DLT-VATS lobectomy revealed no differences, except for the length of hospital stay, which was shorter in the NIVATS group.

In a meta-analysis that included one RCT and seven retrospective reviews involving 970 patients, Xue et al. [61] compared the outcomes of patients undergoing awake lobectomy or segmentectomy with NIVATS or GA. They found that the NIVATS group had a significantly shorter hospital stay, shorter duration of chest drainage, and shorter total time during surgery. Significant differences in the number of lymph nodes removed, operative time, or the drainage volume were not observed. The rate of postoperative complications was similar in both groups. Interestingly, patients with early-stage lung cancer (stages I and II) who underwent awake anatomic lung resection had significantly fewer post-operative complications than patients who underwent GA.

A meta-analysis performed by Zhang et al. [45] included 14 randomized controlled trials comparing NIVATS to DLT-VATS procedures in 1426 patients (707 NIVATS, 719 DLT-VATS). The authors defined minor (bullectomy, wedge resection, sympathectomy, talc pleurodesis, pleural biopsy), moderate (NUSS surgery, lung volume reduction surgery) and major (lobectomy, segmentectomy) thoracic procedures.

Excluding complex anatomic resections, they found a significantly shorter length of hospital stay in NIVATS patients. However, because of the large heterogeneity of patients in the included studies, comparability between the NIVATS and GA cohorts was compromised. Interestingly, not only complications related to intubation but also the overall incidence of complications, except for postoperative atelectasis and pulmonary infection, were lower in the NIVATS group.

Reported benefits of tracheal resections in non-intubated patients include improved airway clearance without the interference of an endotracheal tube, which technically facilitates anastomosis. In addition, blood oxygen saturation (SpO_2_) has been shown to be more stable during the procedure [28].

### Disadvantages

In addition to benefits related to reduced anaesthesia time, length of hospital stay, blood loss, and postoperative pain, problems associated with NIVATS have also been noted.

#### Obese patients

A body mass index (BMI) greater than 30 kg/m^2^ is considered an exclusion criterion for non-intubated surgical access [62, 63]. Obese patients have the anatomical disadvantage of a higher mediastinum- to-chest ratio and a higher position of the diaphragm due to increased intra-abdominal pressure, which may be associated with a potential risk of respiratory depression [64].

In their two centre case series comparing single incision NIVATS in Taiwan (170 patients) and Spain (18 patients), Wang et al. [64] included patients with a BMI up to 35 kg/m^2^. The mean BMI in their study was 22.5 ± 2.8 in Taiwan and 24.8 ± 6.1 in Spain, with a significantly higher BMI in the Spanish cohort (p = 0.005). They did not investigate postoperative outcome stratified by BMI. No ICU admissions were observed in patients with a BMI > 30 kg/m^2^. In one patient (BMI = 30.7 kg/m^2^) anaesthesia had to be switched due to excessive mediastinal movement.

To evaluate the feasibility of awake lung cancer resections (lobectomy, segmentectomy, wedge resection ± lymph node dissection) in patients with a BMI > 25 kg/m^2^ Wu et al. [65] performed a propensity score matching analysis of 48 couples undergoing lung cancer resection in NIVATS and DLT-VATS technique. With a mean BMI of 26.92 kg/m^2^, including only seven obese patients with a BMI > 30 kg/m^2^. They compared short-term outcomes such as operative time, anaesthesia time, intraoperative bleeding volume and length of hospital stay between groups and found no significant difference between the groups. They emphasized that their results applied to moderately overweight patients (BMI between 25 and 30 kg/m^2^) but not to obese patients (> 30 kg/m^2^).

To facilitate surgery in this group of patients with a high rigid diaphragm and/or obesity Kurihara et al. [26] suggested CO_2_-insufflation (< 5 mmHg pressure) for thoracoscopic lung biopsies.

#### Extensive coughing—handling the vagus nerve

Severe cough can interfere with the surgical procedure and increase perioperative risks for the patient. The cough is usually caused by strain on the vagus nerve, which is often induced during manipulation of the pulmonary hilus. Kurihara et al. [26] believe that the NIVATS approach is not appropriate for major lung resections involving the hilum because it may result in significant vagal stimulation leading to cough reflexes [41]. Impairment of the view of the surgical field due to coughing, poor manual surgical control and patient movement were the major technical difficulties cited by 39% of participants in a survey on NIVATS among members of the German Society of Thoracic Surgeons [10] and 59% among members of the ESTS [5].

Xiang et al. [66] compared three groups of 40 patients each (DLT-VATS vs. NIVATS/ICB vs. NIVATS/PVB), who underwent uniportal thoracoscopic wedge resection. In both NIVATS groups, blockade of the vagus nerve was achieved by injecting 10 ml of a mixture of 1% lidocaine and 0.375% ropivacaine near the nerve. In addition, 10 ml of 2% lidocaine was sprayed on the visceral pleura and lung surface. Xiang et al. [66] did not find statistically significant differences within the surgical field between the different groups. According to the authors, the sprayed lidocaine decreases the surface tension, which facilitates lung collapse and blocks the vagus nerve to interrupt the hump reflex and provide a clear and stable surgical field. This technique was also successfully applied by Jiang et al. [7] for thoracoscopic carinal (4 patients) and tracheal (14 patients) resections.

Effective blockade of the vagus nerve requires infiltration of the LA near the nerve at the level of the inferior trachea for right-sided surgery and at the level of the aortopulmonary window for left-sided surgery [18]. Alternatively, 2% lidocaine can be administered endobronchially [46]. This blockade usually lasts for three hours and can be repeated for longer procedures [42, 67]. Some authors advocate inhalation of nebulized 2% lidocaine for 30 min before surgery [41] to reduce the cough reflex, hyperpnea and hypercapnia. A possible adverse effect of the vagus block is a transient recurrent nerve paralysis, which is observed mainly on the left side, where the vagus nerve is sometimes difficult to see, and the LA is injected into the aortopulmonary window [68].

Vagus block becomes even more important when assessing complete lymph node dissection at the hilus in NIVATS and DLT-VATS patients.

#### Atelectasis and postoperative lung expansion

In a propensity score-matched analysis of 119 pairs undergoing lobectomy Lan et al. [69] reported that the incidences of atelectasis, pulmonary exudation, and pleural effusion on postoperative chest radiographs was significantly higher in the NIVATS group. The authors hypothesized that lung expansion is more efficient by direct inflation via the endotracheal tube than asking the patient to cough. Lan et al. [69] also noted that the observed higher rate of the above-mentioned complications could be explained by patient selection, as only lobectomies were included. The authors concluded that secondary infections caused by intraoperative atelectasis could lead to increased exudation [69]. Atelectasis may worsen the shunt fraction and thereby increase the risk of hypoxemia [49]. In order to avoid prolonged atelectasis and to reduce airflow in the corresponding parenchyma a Fogarty balloon may be placed in the targeted lobe bronchus via bronchoscopy before surgery [70]. Rocco et al. [70] described this technique in a case report in 2010 performed before a non-intubated uniportal wedge resection to facilitate atelectasis. However, this technique has not been widely used since then. Another possibility to avoid or attenuate intra-/postoperative atelectasis in NIVATS is to apply positive end-expiratory pressure (PEEP) [47], non-invasive ventilation (NIV) or continuous positive airway pressure (CPAP), respectively. This may be continued postoperatively to decrease the above-mentioned complications. Postoperative PEEP may be adjusted at 5 cm H_2_O or in discussion with the thoracic surgeon to avoid strain on the sutures. If atelectasis occurs on the non-operated lung due to an accidentally opening of the contralateral pleura by the surgeon, a small lumen chest tube can be inserted to re-expand the lung [46].

#### Conversion to GA/tracheal intubation

There are numerous reasons for conversion to a tracheal intubation including intraoperative bleeding, excessive mediastinal movement, massive pleural adhesion, constant uncontrollable cough, unsatisfactory pulmonary collapse, persistent hypoxemia, tachypnoea, poor pain control or panic attacks [6, 41, 64]. Overall conversion rates ranged between 2.8 and 10.0% [59].

A successful NIVATS procedure requires a well-established team of surgeons, anaesthesiologists, and nurses. An inexperienced team and high staff turnover during the procedure can complicate awake thoracic procedures [36, 41, 71, 72].

## NIVATS in primary spontaneous pneumothorax

In 1997, Nezu et al. [73] performed NIVATS blebectomies under LA in 34 patients with primary spontaneous pneumothorax (PSP). All patients but one (91%) stayed free of recurrence. Only minor postoperative complications were observed in three patients, which could be managed conservatively. Two patients had persistent air leak and one patient had transient atelectasis. Compared with 38 patients treated conservatively with DLT-VATS blebectomies during the same period, patients treated with NIVATS had a significantly shorter length of hospital stay.

The first RCT to investigate NIVATS in PSP was performed by Pompeo et al. in 2007 [32]. They randomly assigned 43 of 49 eligible PSP patients to either triportal DLT-VATS (21 patients) or NIVATS/TEA (22 patients) bullectomy with pleural ablation and evaluated the technical feasibility, patient satisfaction, short term outcome, 12 months recurrence rate and hospital costs. Six patients declined randomization and requested DLT-VATS. Technical feasibility was comparably, and operative time did not differ in the two groups. However, anaesthesia time, PACU time, total operating room time, length of hospital stay and costs were significantly in favour of patients treated with NIVATS. The 12 months recurrence rate was similar in both groups. One NIVATS patient and two DLT-VATS patients suffered recurrence of PSP within 1 year.

Mineo et al. [8] report excellent outcomes in their NIVATS/TEA case series published in 2016. They analysed 69 patients treated with triportal bullectomy combined with mechanical pleurodesis and 44 patients treated with uniportal stapling of the target area without removal of lung tissue. Mineo et al. concluded that a major advantage of the awake procedure is the potential for intraoperative cooperation with the patient which facilitates identification of air leaks.

To date NIVATS has not been officially recommended by the German Guidelines for the diagnosis and treatment of PSP (last update in 2018) [74] nor the British Thoracic Society Guidelines on Pleural Diseases (update expected for December 2022) [75]. A 2015 survey conducted by the European Society of Thoracic Surgeons found that only 11% of thoracic surgeons in Europe had performed NIVATS blebectomy or a combination blebectomy/pleurectomy for the treatment of PSP [5].

It is anticipated that as thoracic surgeons become more experienced in non-intubated surgery, NIVATS will become more important in the treatment of PSP because air leaks can be better visualized intraoperatively due to patients’ active breathing, and because the hospital stay is shorter, costs are lower, and short- and long-term outcomes are similar.

## NIVATS in diagnosing pleural effusion and pleural disease

In 2002, Migliore et al. [76] published a larger case series on diagnostics and treatment of pleural effusion and pleural disease using NIVATS. NIVATS/LA was performed in 43 patients to evaluate pleural effusion (39 patients), or unexplained pleural thickening (4 patients). One patient required DLT and minithoracotomy because of intraoperative bleeding. Eight patients developed hyperpyrexia, three hypotension, and two atrial fibrillations postoperatively.

Numerous studies have now demonstrated that NIVATS is safe in the diagnoses of pleural disease and the treatment of pleural effusion. Katlic et al. [77] reported on 244 patients who were safely operated on with NIVATS for pleural effusion. Talc poudrage was performed in 184 of these patients. None of the patients required thoracotomy or intubation. In 2018, McDonald et al. [78] compared the safety and efficacy of therapeutic workup of pleural effusions using NIVATS and DLT-VATS in an outpatient setting at a tertiary thoracic surgery centre in Canada. Seventy-eight patients were treated with NIVATS, and 99 patients were treated with DLT-VATS. Diagnostic yield was similar in both patient cohorts (NIVATS: 93.6% vs. DLT-VATS: 96%). The observed rate of minor complications was 17.9% in the NIVATS group and 16.2% in the DLT-VATS group of patients. Major complications (e.g., haemorrhage, alveolar-pleural fistula, hemodynamic instability, or respiratory failure) occurred in 2.6% of the NIVATS patients and in 4% of DLT-VATS patients. However, NIVATS was associated with shorter length of hospital stay (NIVATS: mean 0 days vs. DLT-VATS: 3 days) and lower procedure-related costs.

A survey conducted by the German Society of Thoracic Surgeons [10] and the European Society of Thoracic Surgeons [5] found that 87% of thoracic surgeons in Germany and 98% of thoracic surgeons in Europe have already performed NIVATS pleural biopsies to investigate recurrent pleural effusions and pleural disease.

## NIVATS in ILD patients

When the diagnosis of ILD cannot be confirmed by high-resolution computed tomography (HRCT) surgical lung biopsy (SLB) is recommended to make a definitive diagnosis [79].

Reported morbidity and mortality rates for patients undergoing SLB for undetermined ILD in DLT-VATS technique range from 5.8 to 14.7% and 1.4 to 4%, respectively [80, 81]. These findings prompted Pompeo et al. [19] in 2013 to conduct a pilot feasibility study of SLB in NIVATS technique in ILD patients. In 30 patients, technical feasibility was assessed by consensus between the surgeon and anaesthesiologist and was rated excellent in 20 patients and good in nine patients. They reported no postoperative mortality and only one patient developed postoperative fever and pulmonary atelectasis (3.3%) which resolved spontaneously.

Kurihara et al. [26] reported similar findings in their retrospective study of 44 ILD patients who underwent SLB (NIVATS: 15 vs. DLT-VATS: 29). They found that ILD worsened significantly more often in the GA group. One patient died after GA-SLB due to acute ILD exacerbation. A Cox proportional hazard analysis revealed that older patients, longer anaesthesia time and GA were independent predictors of worsening ILD after SLB. The authors hypothesized that avoiding atelectasis would improve the outcome of ILD patients undergoing SLB.

Guerrera et al. [82] assigned 100 patients with ILD to either DLT-VATS or NIVATS SLB after multidisciplinary discussion (thoracic surgeon, anaesthesiologist, pneumonologist). NIVATS SLB was performed in 66 and DLT-VATS in 34 patients using biportal (55%), uniportal (35%) or triportal (10%) techniques. Propensity score matching analysis showed that NIVATS SLB was associated with lower postoperative morbidity and less frequent unexpected ICU admission as well as shorter operative and anaesthesia times and shorter chest tube indwelling time.

Pompeo et al. [83] performed a multicentre retrospective analysis to evaluate the short-term postoperative outcomes of SLB using the uni- and multiport NIVATS technique. They observed no ILD exacerbations and no postoperative mortality. They attributed the low overall morbidity rate of 7.1% (bronchopleural fistulae, pneumonia, atelectasis, anaemia, and gastric bleeding) to the fact that patients were not exposed to high oxygen concentrations and lung overdistension with the NIVATS technique. Peng et al. [84] also reported no 30-day mortality in 43 ILD patients who underwent SLB using the uniportal NIVATS technique. In all patients, the chest tube could be removed while the patient was still in the operating room. A propensity score matching analysis by Grott et al. [85] showed faster postprocedural recovery of patients after NIVATS with similar procedure-related complications compared with DLT-VATS.

However, a 2019 review by Amundson et al. [86] found acute exacerbation of ILD (AE-ILD) after various procedures (SLB, lung resections, non-lung surgery or transbronchial cryoprobe lung biopsy). NIVATS procedures were not included. Rates of AE-ILD ranged from 1% after wedge resections to 15% after lung cancer resections or 2.3% after cryoprobe biopsy. According to the authors, hyperoxia, one-lung ventilation, undiagnosed infections (viral or bacterial) and aspiration events can trigger postprocedural AE-ILD. Hyperoxia can lead to the generation of reactive oxygen, which then damages alveolar cells. In addition, high concentrations of inhaled oxygen (FiO_2_) can cause irritation of the tracheobronchial tree, which promotes atelectasis and leads to secretion retentions. In addition, overdistension of the dependent lung and changes in cytokines may contribute to AE-ILD. However, the exact mechanism and contribution of each alteration to develop AE-ILD remain unknown.

Kim et al. [87] reviewed three studies for SLB in NIVATS technique. No postprocedural mortality and only minor morbidity were described, ranging from 3.3 to 7.0% including ARDS, pneumonia, or atrial fibrillation. Table 2 provides an overview of the included studies on ILD patients who underwent NIVATS SLB.

**Table 2:** Interstitial lung disease (ILD) patients undergoing surgical lung biopsy (SLB)

Author	Year	Study type	Analgesia	No. of Pts.	Results	Diagnostic yield (%)
Pompeo et al. [19]	2013	PFS	EC: 20 IB: 10	NIVATS: 30	Technical feasibility correlated with DLCO Excellent in 20 Good in 9 Satisfactory in 1 Postoperative morbidity 3.3%	97
Peng et al. [84]	2017	CS	LA	NIVATS: 43	Chest tube removal on operating table Postoperative morbidity: 7%, no death	88.4
Pompeo et al. [83]	2019	CS (MC)	IB: 84 EC: 28	NIVATS: 112	Perioperative morbidity: 7.1%, no death Mean hospital stay: 2.5 days	96
Kurihara et al. [26]	2020	CS	EC	NIVATS: 15 DLT-VATS: 29	Reduced surgical time in NIVATS: 32.5 vs. 50.8 (min) Shorter length of hospital stay in NIVATS: 1 vs. 10 (days) Significant more ILD worsening in DLT-VATS group (one death)	100
Kim et al. [87]	2020	REV (3 studies)	1. EC: 20 1. IB: 20 2. IV: 43 3. EC: 10	1. NIVATS: 40 2. NIVATS: 43 3. NIVATS: 10	EC/IB mainly used for pain control IB has benefit in operation time and hospital stay Low morbidity rates: 3.3–7.0%	82.5–100
Guerrera et al. [82]	2021	PSM	EC + LA	NIVATS: 66 DLT-VATS: 34	Lower postoperative morbidity in NIVATS 3.0% vs. 20.6% Shorter length of hospital stay in NIVATS 3.1 vs. 6.7 (days) Reduced surgical time in NIVATS 38 vs. 77 (min) Reduced anaesthesiologic time in NIVATS 97 vs. 132 (min)	73
Grott et al	2022	PSM	EC	NIVATS: 40 DLT-VATS: 40	Faster postprocedural recovery after NIVATS Postoperative acute exacerbation of ILD similar between groups	98.75

*No. of Pts.* number of patients, *PFS* prospective feasibility study, *EC* epidural catheter, *IB* intercostal block, *NIVATS* non-intubated video assisted thoracic surgery, *DLCO* diffusion for carbon monoxide, *CS* case series, *LA* local anesthesia, *MC* multicenter, *DLT-VATS* double-lumen tube—video assisted thoracic surgery, *PSM* propensity score matching, *REV* review, *IV* intravenous anesthesia, *ILD* interstitial lung disease

## NIVATS in surgical oncology for lung cancer

Whenever a new (surgical) technique is introduced, its noninferiority to the state-of-the-art technique must be demonstrated. NIVATS for patients with NSCLC must result in comparable long-term survival to other established DLT-VATS and open surgical procedures. Systematic lymph node dissection plays a critical role in patients, determining prognosis and further adjuvant treatment. For the establishment of the NIVATS technique, it is of great importance to demonstrate that lymph node dissection using the NIVATS technique is as safe and effective and provides the same results as DLT-VATS or open surgical procedures. To date, NIVATS lobectomy has been proposed for patients with lung cancer at an early tumour stage (I + II), tumour size less than 6 cm, without tumour infiltration of the main bronchus and without evidence of N2 lymph nodes [40, 63, 88].

### Short-term outcomes

Ali et al. [89] performed a review of five studies (three cases series, one randomized control trial and one meta-analysis) comparing NIVATS with DLT-VATS lobectomy for patients with lung cancer. All studies consistently showed faster recovery of operated patients from anaesthesia and faster ability to feed and mobilize, resulting in shorter hospital stay after NIVATS with comparable postoperative complication rates (including bronchopleural fistula, pneumonia, atrial fibrillation). A meta-analysis by Prisciandaro et al. [90] included three retrospective cohort studies with a total of 204 patients with NSCLC comparing outcomes after NIVATS and DLT-VATS lobectomy. No statistically significant differences were demonstrated comparing the two surgical techniques.

### Long-term outcomes/survival

There is limited information on the long-term survival of NSCLC patients operated on by NIVATS. Wang et al. [91] compared patients with early-stage NSCLC operated on by either DLT-VATS or NIVATS technique. Ninety-seven lobectomy pairs (NIVATS vs. DLT-VATS) were compared after propensity score matching. The groups were comparable in terms of histology (adenocarcinoma 97% vs. 92%), tumour size (19.8 mm vs. 20.0 mm), tumour stage (IA1: 13% vs. 16%; IA2: 47% vs. 38%; IA3: 30% vs. 32%; IB: 10% vs. 14%), and anatomic location. The mean follow-up time was 74.2 months. No significant differences were observed between the NIVATS and DLT-VATS groups in terms of locoregional recurrence (9.3% vs. 16.5%) or distant metastases (10.3% vs. 18.6%). Recurrence-free survival (85.6% vs. 74.2%) and overall survival at 5 years (97.9% vs. 93.8%) also did not differ significantly between the groups. Zheng et al. [92] also performed a propensity score matching analysis of 200 patients with NSCLC after NIVATS and DLT-VATS surgery and examined disease-free survival and overall patient survival. In addition, they compared survival of their NIVATS/DLT-VATS cohorts with an open approach in a 1:1:1 propensity score matching. The NIVATS technique was associated with a better 3-and 5-year overall- and disease-free survival. The authors cited a reduced need for opioids and the use of regional anaesthesia in combination with intravenous anaesthesia as possible explanations for the positive effect on long-term survival. Another positive effect, according to the authors, could be the avoidance of a pro-metastatic effect of volatile anaesthetics. Zheng et al. [92] note that the median follow-up time of 4.78 years of their NIVATS patients was not long enough to calculate median survival.

### Lymph node yield

Chen et al. [40] observed no statistically significant difference in the lymph node yield in their comparative case series of 30 NS patients each undergoing lobectomy with NIVATS or DLT-VATS for lung cancer (NIVATS: 13.8 ± 6.0 vs. DLT-VATS: 14.0 ± 6.0).

The number of resected lymph nodes in geriatric patients (> 65 years) undergoing lobectomy for non-small cell lung cancer was also studied in a series by Wu et al. [68] and showed no statistically significant difference. The number of dissected lymph nodes was 13.1 ± 7.7 in NIVATS and 15.5 ± 8.1 in DLT-VATS. The authors reported that despite vagal blockade, a cough reflex was elicited in some patients in the NIVATS cohort during dissection of the infracarinal lymph nodes due to involvement of the contralateral main stem bronchus.

Wu et al. [65] reported that the total number of resected lymph nodes was slightly lower in the DLT-VATS group (5 ± 10 vs. 9 ± 11). They performed propensity score matching of patients undergoing lung cancer surgery and included patients after lobectomies, segmentectomies and wedge resections. Dissection of the N1 and N2 lymph nodes was routinely performed in all patients. No difference was observed between the two groups in the number or location of resected lymph nodes.

Guo et al. [93] also observed no difference in the number of resected lymph nodes in patients with NSCLC who underwent parenchyma-sparing resection by NIVATS or DLT-VATS (8.06 ± 6.22 vs. 8.02 ± 4.31). Liu et al. [42] also failed to demonstrate a statistically significant differences after propensity score matching in the number of lymph nodes removed in 116 patients after lobectomy- (17.2 ± 9.1 vs. 15.7 ± 9.5) and 20 patients after segmentectomy (7.8 ± 5.4 vs. 6.4 ± 5.3). Interestingly, significantly fewer patients developed pleural effusion postoperatively after NIVATS surgery. The authors attributed this to avoidance of inhalation anaesthetics and muscle relaxants because of their negative effects on intestinal absorption and metabolism. Liu et al. [54] also observed lower inflammatory cytokine levels in the blood of patients after NIVATS surgery and suggested that this also counteracts the development of pleural effusion.

In a case series by Ahn et al. [63] the median number of dissected lymph nodes in patients undergoing uniportal NIVATS lobectomy (21 patients) or segmentectomy (six patients) for lung cancer was 11.33 ± 7.0. Wang et al. [64] reported a median of five dissected lymph nodes (range 1–18) in their two-centre cohort of 188 patients who underwent either lobectomy, segmentectomy or wedge resection. Unfortunately, the authors did not examine the lymph node yield specifically to the surgical procedure.

Only AlGhamdi et al. [88] reported on a significantly lesser lymph node yield in their NIVATS patients (12.6 ± 6.0 vs. 18.0 ± 7.4).

A significantly lower number of N2 station lymph nodes (2.63 ± 1.11 vs. 3.03 ± 1.18) in their NIVATS cohort was also noted by Zheng et al. [92] but this was not reflected in the total amount of N2 lymph nodes harvested between groups (10.91 ± 8.35 vs. 12.04 ± 7.83). Table 3 provides an overview of the included studies of NIVATS in surgical oncology for lung cancer and their lymph node yield.

**Table 3:** Non-intubated video-assisted thoracic surgery (NIVATS) in surgical oncology

Author	Year	Study type	No. of Pts.		Procedure	Lymph node yield			Postoperative morbidity (%)/mortality (%)		
			NIVATS	DLT-VATS		NIVATS	DLT-VATS	p-value	NIVATS	DLT-VATS	p-value
Chen et al. [40]	2011	CS	30	30	Lobectomy	13.8	14.0	0.915	10/0	33/0	n.a
Wu et al. [68]	2013	CS	36	48	Lobectomy	13.1	15.5	0.133	25/0	35/0	0.489
Guo et al. [93]	2016	CS	48	92	Segmentectomy	8.06	8.02	0.969	8.3/0	15.2/0	0.248
Liu et al. [42]	2016	PSM	146	146	Lobectomy	15.7	17.2	0.223	10.3/0	8.6/0	0.654
					Segmentectomy	6.4	7.8	1	15/0	15/0	1
Wang et al. [64]	2017	CS	188	n.a	Wedge resection/lobectomy/segmentectomy	5	n.a	n.a	8.5/0	n.a	n.a
AlGhamdi et al. [88]	2018	CS	31	31	Lobectomy	12.6	18.0	0.003	20/0	20/0	0.862
Ahn et al. [63]	2018	CS	40	n.a	Lobectomy/segmentectomy/wedge resection	11.3	n.a	n.a	17.5/0	n.a	n.a
Wu et al. [65]	2020	PSM	48	48	Lobectomy/segmentectomy/wedge resection	9	4	0.14	13/0	4/0	0.27
Zheng et al. [92]	2021	PSM	200	200	Lobectomy	N2: 10.91	N2: 12.04	0.162	n.a./0	n.a./0	n.a

*No. of Pts.* number of patients, *NIVATS* non-intubated video-assisted thoracic surgery, *DLT-VATS* double lumen tube video-assisted thoracic surgery, *CS* case series, *n.a.* not available/not mentioned, *PSM* propensity score matching

## General considerations on current literature on NIVATS (in lung cancer)

The literature comparing NIVATS with DLT-VATS in thoracic surgery consists mainly of selected case series and single centre experience reports, such as published by Tacconi et al. [2] in 2016. Most studies lack control groups and compare different surgeries [69]. Caution should be exercised when interpreting these studies because of the lack of sample size estimation, which affects statistical power [67]. Additional bias in the data collected may be explained by the fact that NIVATS surgeries are usually performed by experienced surgeons, anaesthesiologists, and operating room nurses in centres with great expertise and a large case volume [60].

Specific literature on NIVATS in lung cancer patients is even scarcer and consists only of retrospective observational studies and single case series. The total number of patients operated on by NIVATS for anatomical lung resections remains small. Larger randomized trials are not available. However, published data strongly suggest that short-term outcomes are not significantly different from intubated patients and that NIVATS is technically feasible and safe and noninferior to DLT-VATS. Most studies do not report differences in the yield of resected lymph nodes [40, 65, 68, 93]. We found one study reporting long-term data comparing the two different procedures that showed no oncological disadvantage for NIVATS [91], whereas another study demonstrated superiority of NIVATS over DLT-VATS in terms of long-term survival [92]. The lack of long-term data may be due to the fact that NIVATS lobectomy has only been performed for about a decade with the first series reported by Chen et al. [40] in 2011.

In addition, selection bias could arise from highly selected patients due to the strict inclusion criteria for NIVATS procedures such as low BMI, no evidence of N2 disease or bronchial involvement.

## Conclusion

Despite the small number of studies of NIVATS, published data suggest that NIVATS is safe and appropriate for selected patients. Prospective studies are urgently needed to validate the NIVATS technique. The major benefits for patients operated on with NIVATS are faster postoperative recovery, less issues with digestion, less pain, and shorter length of hospital stay. NIVATS can be safely used for minor, moderate, and major thoracic procedures. NIVATS appears to be noninferior to DLT-VATS in patients with early-stage lung tumour [33]. Especially patients with ILD seem to benefit particularly from the NIVATS technique. As the NIVATS technique becomes more widely used, more evidence for its use will be generated in the coming years, which is urgently needed to validate the method.

## Data Availability

Data sharing is not applicable to this article as no datasets were generated or analysed during the current review. Literature/cited references were searched and found via PubMed.

## Abbreviations

AE-ILD: Acute exacerbation of interstitial lung disease
ATS: Awake thoracic surgery
AVATS: Awake video assisted thoracic surgery
BIS: Bispectral index sensor
BMI: Body mass index
CEA: Cervical epidural anaesthesia
CPAP: Continuous positive airway pressure
DGT: German Society of Thoracic Surgery (Deutsche Gesellschaft für Thoraxchirurgie)
DLT: Double lumen tube
DLT-VATS: Double lumen tube video assisted thoracic surgery
ESPB: Erector spinae plane block
ESTS: European Society of Thoracic Surgeons
GA: General anaesthesia
hs-CRP: High-sensitivity C-reactive protein
ICB: Intercostal (nerve) block
ILD: Interstitial lung disease
LA: Local anaesthetic
MAC: Minimal alveolar concentration
MACTS: Monitored anaesthesia care thoracic surgery
MAP: Mean arterial pressure
NITS: Non-intubated thoracic surgery
NIV: Non-invasive ventilation
NIVATS: Non-intubated video assisted thoracic surgery
NSCLC: Non-small cell lung cancer
OLV: One-lung ventilation
PACU: Post anaesthesia care unit stay
PALI: Postoperative acute lung injury
PECS: Pectoralis nerve block
PEEP: Positive end-expiratory pressure
PPC: Postoperative pulmonary complications
PSM: Propensity score matching
PSP: Primary spontaneous pneumothorax
PVB: Paravertebral block
RCT: Randomized control trial
SAPB: Serratus anterior plane block
SLB: Surgical lung biopsy
SOP: Standard operating procedure
SV-VATS: Spontaneous-ventilation video-assisted thoracoscopic surgery
TEA: Thoracic epidural anaesthesia
TNF-α: Tumour necrosis factor α
VATS: Video assisted thoracic surgery
